# Construct Validity and Factor Structure of the Pittsburgh Sleep Quality Index and Epworth Sleepiness Scale in a Multi-National Study of African, South East Asian and South American College Students

**DOI:** 10.1371/journal.pone.0116383

**Published:** 2014-12-31

**Authors:** Bizu Gelaye, Vitool Lohsoonthorn, Somrat Lertmeharit, Wipawan C. Pensuksan, Sixto E. Sanchez, Seblewengel Lemma, Yemane Berhane, Xiaotong Zhu, Juan Carlos Vélez, Clarita Barbosa, Asterio Anderade, Mahlet G. Tadesse, Michelle A. Williams

**Affiliations:** 1 Department of Epidemiology, Harvard School of Public Health, Boston, MA, United States of America; 2 Department of Preventive and Social Medicine, Faculty of Medicine, Chulalongkorn University, Bangkok, Thailand; 3 College of Public Health Sciences, Chulalongkorn University, Bangkok, Thailand; 4 School of Nursing, Walailak University, Nakhon Si Thammarat, Thailand; 5 Universidad San Martin de Porres, Lima, Peru; 6 Addis Continental Institute of Public Health, Addis Ababa, Ethiopia; 7 Centro de Rehabilitación Club de Leones Cruz del Sur, Punta Arenas, Chile; 8 Department of Mathematics & Statistics, Georgetown University, Washington, DC, United States of America; Peking University, China

## Abstract

**Background:**

The Pittsburgh Sleep Quality Index (PSQI) and the Epworth Sleepiness Scale (ESS) are questionnaires used to assess sleep quality and excessive daytime sleepiness in clinical and population-based studies. The present study aimed to evaluate the construct validity and factor structure of the PSQI and ESS questionnaires among young adults in four countries (Chile, Ethiopia, Peru and Thailand).

**Methods:**

A cross-sectional study was conducted among 8,481 undergraduate students. Students were invited to complete a self-administered questionnaire that collected information about lifestyle, demographic, and sleep characteristics. In each country, the construct validity and factorial structures of PSQI and ESS questionnaires were tested through exploratory and confirmatory factor analyses (EFA and CFA).

**Results:**

The largest component-total correlation coefficient for sleep quality as assessed using PSQI was noted in Chile (r = 0.71) while the smallest component-total correlation coefficient was noted for sleep medication use in Peru (r = 0.28). The largest component-total correlation coefficient for excessive daytime sleepiness as assessed using ESS was found for item 1 (sitting/reading) in Chile (r = 0.65) while the lowest item-total correlation was observed for item 6 (sitting and talking to someone) in Thailand (r = 0.35). Using both EFA and CFA a two-factor model was found for PSQI questionnaire in Chile, Ethiopia and Thailand while a three-factor model was found for Peru. For the ESS questionnaire, we noted two factors for all four countries

**Conclusion:**

Overall, we documented cross-cultural comparability of sleep quality and excessive daytime sleepiness measures using the PSQI and ESS questionnaires among Asian, South American and African young adults. Although both the PSQI and ESS were originally developed as single-factor questionnaires, the results of our EFA and CFA revealed the multi- dimensionality of the scales suggesting limited usefulness of the global PSQI and ESS scores to assess sleep quality and excessive daytime sleepiness.

## Introduction

Sleep insufficiency, caused by societal factors such as round-the-clock access to technology and work schedules is recognized as an important global public health problem [1]–[3] affecting some 45% of the world's population [4]. Individuals experiencing poor sleep quality and insomnia symptoms have increased risks of chronic disorders such as cancer, hypertension, diabetes, psychiatric symptoms, cognitive impairment and obesity, as well as increased mortality, and reduced quality of life and productivity [2], [5]. Moreover, daytime sleepiness has been shown to contribute to motor vehicle crashes, industrial disasters, and medical and other occupational errors [2]. A recent poll by the National Sleep foundation in the US has found that 43% of Americans between the ages of 13 and 64 years suffer from sleep insufficiency [6]. A growing body of evidence, primarily from populations in high-income countries, shows a decreasing trend in average sleep duration and a higher prevalence of insomnia and other sleep disorders [3], [7]–[9]. In recognition of the importance of sufficient sleep to population health, organizations such as the World Association of Sleep Medicine [4] have begun to mobilize health professionals to deliver messages about the importance of healthy sleep habits to populations worldwide.

Furthermore, public health agencies such as the US Centers for Disease Control and Prevention [10] have expanded surveillance of sleep-related behaviors in recent years [11]. Such efforts, in part, have led to the documentation of the high prevalence of unhealthy sleep behaviors and self-reported sleep-related difficulties among US adults. However, comparable information among adults from low- and middle-income countries (LAMICs), particularly young adults are sparse [12]. Additionally, despite routine use among adults and among individuals from North American, European and some Asian countries, the psychometric properties and factor structure of sleep questionnaires such as the Pittsburgh Sleep Quality (PSQI) and Epworth Sleepiness Scale (ESS) have not been adequately assessed among young adults, particularly those from diverse geographic, racial and ethnic backgrounds [13]–[17]. The PSQI and ESS are questionnaires commonly used to assess sleep quality and excessive daytime sleepiness [13], [16]. The psychometric properties of the PSQI and ESS have been examined in clinical and population-based studies; and investigators have reported good internal consistency, concurrent validity and discriminative validity for the questionnaires [13]–[17]. However no study has evaluated their cross-cultural validity and equivalence in LAMICs. Evaluating the factor structure of questionnaires when applied in new context or cultural background has been one of the widely used methods for assessing psychometric properties. Therefore, using data from a large multi-country study involving 8,481 college students across three continents, we sought to evaluate the psychometric properties of the PSQI and ESS. Further, given that there appears to be no consensus about the best representation of the factor structure of the PSQI [18]–[20] and ESS [21], we also investigated the factors structure of the questionnaires and assessed possible differences across study populations.

## Methods and Materials

A multi-country survey of college students was conducted in Chile, Ethiopia, Peru, and Thailand between November, 2010 and May, 2011. Methodological details of the surveys have been published elsewhere [22]–[25]. Briefly, flyers were posted in each campus to invite participants to the study. Students who expressed an interest in participating were invited to meet in a large classroom or an auditorium where they were informed about the purpose of the study. Students consenting to participate were invited to complete a self-administered anonymous individual survey. Vision impaired students and those who could not read the consent and questionnaire forms were not eligible to participate. Those enrolled in correspondence, extension, or night school programs were not included as well since their experience might be different from regular time students.

The sample consists of 8,481 college students ≥18 years of age from Chile (N = 830), Ethiopia (N = 2,230), Peru (N = 2,581), and Thailand (N = 2,840) using a common research protocol. The surveys in each country were conducted at a time that does not conflict with exam periods. In each country, the questions were translated from English into the local language, following the WHO translation guidelines for assessment instruments. This included a forward translation, a targeted back-translation, and review by a bilingual expert group [26]. Ethical approvals from the following institutional review boards were obtained prior to the commencement of the data collection procedures: Centro de Rehabilitación Club de Leones Cruz del Sur, Punta Arenas, Chile; Addis Continental Institute of Public Health and Gondar University, Ethiopia; Dos de Mayo Hospital and Universidad Nacional Mayor de San Marcos in Lima, Peru; Faculty of Medicine Chulalongkorn University, Walailak University, Thailand, and the University of Washington, USA. The Harvard School of Public Health Office of Human Research Administration, USA, granted approval to use the anonymised datasets for analysis.

### Data Collection and Variables

As noted above, a self-administered questionnaire was used to collect information for this study. The questionnaire ascertained demographic information including age, sex, and education level and behavioral risk factors including physical inactivity, cigarette smoking, alcohol consumption and the consumption of energy drinks, caffeinated beverages, and other stimulants. According to the World Health Organization (WHO) protocol measurements of the student's height and weight, were also collected by research nurses after the questionnaire was completed [27].

### Pittsburgh Sleep Quality Index (PSQI)

The PSQI is a 19-item self-reported questionnaire that evaluates sleep quality over the past month [13]. The PSQI yields seven sleep components related to sleep habits including duration of sleep, sleep disturbance, sleep latency, habitual sleep efficiency, use of sleep medicine, daytime dysfunction due to sleepiness, and overall sleep quality. Each sleep component yields a score ranging from 0 to 3, with 3 indicating the greatest dysfunction [13]. Subsequently, the sleep component scores are summed to yield a global sleep quality score (range 0 to 21) with higher scores indicating poor sleep quality during the previous month. The distribution of the PSQI score by country is presented in **S1 Fig**. Based on prior literature, participants with a global score of >5 were classified as poor sleepers. Those with a score of ≤5 were classified as good sleepers. This classification is consistent with prior studies of college students [28]. The prevalence of poor sleep quality using this published cut-off by country is presented in **S3 Fig**.

### Epworth Sleep Scale (ESS)

The ESS is an 8-item questionnaire designed to measure a person's general level of daytime sleepiness [16] and the capability to stay alert and awake during crucial moments of the day [29]. The 8 items capture an individual's propensity to fall asleep during commonly encountered situations, each measured on a Likert scale from 0 to 3. The distribution of the ESS score by country is presented in **S2 Fig**. The scores for the eight questions are added together to obtain a single total score that ranges from 0 to 24. In adults, an ESS score ≥10 is taken to indicate excessive daytime sleepiness [16]. The prevalence of excessive daytime sleepiness using this published cut-off by country is shown in **S3 Fig**.

### Other Covariates

We defined alcohol consumption as low (<1 alcoholic beverage a week), moderate (1–19 alcoholic beverages a week), and high to excessive consumption (≥20 alcoholic beverages a week) [30], [31]. Other covariates were categorized as follows: age (years), sex, cigarette smoking history (never, former, current) [22], and participation in moderate or vigorous physical activity (no vs. yes); BMI was calculated as weight (in kilograms)/height squared (in square meters). BMI thresholds were set according to the WHO protocol (underweight <18.5; normal, 18.5–24.9; overweight, 25.0–29.9; and obese, ≥30 kg/m^2^) [27].

### Statistical Analysis

We first explored frequency distributions of demographic and lifestyle characteristics of participants in each country. Participants' characteristics were summarized using means (± standard deviation [SD]) for continuous variables and counts and percentages for categorical variables with normal distributions. For variables with non-normal distribution as assessed using the Shapiro–Wilk test, we report median (Interquartile range [IQR]). We assessed the internal consistency of the PSQI and ESS using Cronbach's alpha and item-total correlations. We report the Spearman correlation as a measure of the direction and strength of item-total correlations. Further, we explored the factor structures of the two questionnaires using both exploratory factor analysis [32] and confirmatory factor analysis [33] approaches. Prior to performing EFA, we assessed the suitability of the data for performing factor analysis. This analysis showed that it was appropriate to proceed with factor analysis (Bartlett's test of sphericity, p-value <0.001 for both questionnaires in all four countries; and the Kaiser-Meyer-Olkin measure of sampling adequacy, ranging from 0.724 to 0.801). We conducted the EFA using principal component analysis with orthogonal rotation. We used the scree plot, presenting eigenvalues associated with each factor, to determine factor structure. Factors with eigenvalues >1 were assumed to be meaningful and were retained for rotation. Rotated factor loadings of >0.4 were considered sufficient, while items with factor loadings ≥0.4 on more than one factor were considered cross-loading. To complement our EFA and to evaluate the model fit, we conducted CFA using maximum likelihood estimation approaches. In order to assess model fit, we calculated the Root Mean Square Error of Approximation (RMSEA) along with 95% confidence interval (95% CI), comparative fit index and Tucker-Lewis index. The RMSA is a measure of fit that considers how much error there is for each degree of freedom. The comparative fit index is a widely used measure that compares the model with a baseline model that assumes there is no relationship among the observed indicated variables. Despite the absence of consensus concerning the cut-off for goodness of fit, we elected to use the criteria recommended by Brown [33]. Brown recommended that the following criteria were evidence for reasonably good fit: 1) SRMR values ≤0.08; 2) RMSEA values ≤0.06 or below; 3) comparative fit index ≥0.95. All statistical analyses were performed using Stata version 12.0 software (Statacorp, College Station, TX). The level of statistical significance was set at p-values <0.05 and all tests were two-sided.

## Results

A total of 8,481 college students from four countries participated in the study. **Table 1** shows demographic and lifestyle characteristics of participants stratified by country. The majority of participants in Chile (71.1%), Peru (61.2%) and Thailand (67.4%) were females. In Ethiopia, only 22.7% of participants were female students. Current smoking was reported by approximately 44% of students in Chile, 17% in Peru, 7% in Thailand and 3% in Ethiopia. Moderate alcohol consumption (≥1 drink/month was reported by 79.8% of students in Peru, 34.6% in Chile, 14.8% in Ethiopia and 23.0% in Thailand. Consumption of any caffeinated beverages was reported by the majority of students in Ethiopia (80.4%), Thailand (57.7%), and Chile (54.9). In Peru 40% of students reported consumption of any caffeinated beverages. Ethiopia had the highest proportion of underweight students (39%); whilst Chile had the highest proportion of overweight and obese students (43.6%).

**Table 1:** Demographic and lifestyle characteristics of college students according to county.

	Chile	Ethiopia	Peru	Thailand	
	N = 830	N = 2230	N = 2581	N = 2840	Effect Size**
	n (%)	n (%)	n (%)	n (%)	
Age (years)*	21.9±3.4	21.6±1.7	20.9±2.6	20.3±1.3	
Age (years)					
18–19	221 (26.6)	123 (5.5)	902 (35.1)	811 (28.6)	0.23
20	121 (14.6)	454 (20.4)	339 (13.2)	820 (28.9)	
21	99 (11.9)	627 (28.1)	410 (16.0)	695 (24.5)	
22	111 (13.4)	418 (18.7)	309 (12.0)	371 (13.1)	
≥23	278 (33.5)	608 (27.3)	608 (23.7)	143 (5.0)	
Sex					
Male	240 (28.9)	1700 (77.3)	1002 (38.8)	925 (32.6)	0.40
Female	590 (71.1)	499 (22.7)	1579 (61.2)	1915 (67.4)	
Body Mass Index (kg/m^2^)*	24.9±4.4	19.2±1.7	23.5±4.4	21.6±3.8	
Body Mass Index (kg/m^2^)					
Underweight (<18.5)	10 (1.2)	857 (38.6)	84 (4.3)	474 (16.7)	0.28
Normal (18.5–24.9)	444 (55.2)	1337 (60.2)	1299 (66.8)	1974 (68.6)	
Overweight (25.0–29.9)	227 (28.2)	28 (1.3)	481 (24.7)	287 (10.1)	
Obese(≥30)	124 (15.4)	0 (0)	82 (4.2)	132 (4.6)	
Alcohol Consumption					
<1 drink/month	489 (63.8)	1900 (85.2)	522 (20.2)	2187 (77.0)	0.12
1–19 drinks/month	252 (32.9)	303 (13.6)	1013(39.3)	607 (21.4)	
≥20 drinks/month	13 (1.7)	27 (1.2)	1046 (40.5)	46 (1.6)	
Cigarette Smoking Status					
Never	325 (39.1)	2146 (96.3)	1929 (74.7)	2591 (91.2)	0.30
Former	77 (9.3)	15 (0.5)	220 (8.5)	53 (1.9)	
Current	368 (44.2)	72 (3.2)	432 (16.8)	196 (6.9)	
Consumption of Caffeinated Beverages					
No	365 (45.1)	436 (19.6)	1545 (60.0)	1202 (42.3)	0.30
Yes	433 (54.9)	1789 (80.4)	935 (40.0)	1638 (57.7)	

*mean ± SD (standard deviation).

**Effect size: Cramer's V for chi-square test.

***Numbers may not add up due to missing data for selected variables.

As shown in **Table 2** the Spearman's correlation coefficients of the seven subscales of the PSQI and the PSQI total score showed similar patterns across four countries where all corrected item-total correlations were ≥0.40 except for sleep medication. The largest item-total correlation coefficient was found for sleep quality in Chile (r = 0.71) while the smallest subscale-total correlation coefficient was noted for sleep medication use in Peru (r = 0.28). The lower panel of Table 2 shows results of the correlation coefficients between the eight items of ESS and the ESS total scores by country. The correlation coefficients showed similar patterns across countries where all corrected item-total correlations were >0.30, indicating adequate homogeneity of ESS items in each country. Feeling sleepy while talking to someone in Thailand (r = 0.35) and feeling sleepy while driving in Ethiopia (r = 0.34) had the lowest item–total scale correlation.

**Table 2:** Pittsburgh Sleep Quality Index (PSQI) and Epworth Sleepiness Scale (ESS) subscale-total scale correlations according to country.

Pittsburgh Sleep Quality Index (PSQI)				
	Chile	Ethiopia	Peru	Thailand
N	**830**	**2230**	**2581**	**2840**
Median (IQR)	6 (4,8)	6 (4,8)	6 (4,8)	5 (4, 7)
Sleep Duration	0.62	0.63	0.64	0.58
Sleep Disturbance	0.47	0.46	0.46	0.40
Sleep Latency	0.61	0.53	0.49	0.59
Sleep Efficiency	0.54	0.65	0.42	0.56
Daytime Dysfunction	0.55	0.52	0.57	0.51
Sleep Medication	0.37	0.32	0.28	0.34
Sleep Quality	0.71	0.54	0.63	0.63

Epworth Sleepiness Scale (ESS)				
N	831	2230	2458	2840
Median (IQR)*	7 (5,10)	7 (5,10)	8 (6,11)	8 (5, 10)
Sitting/Reading	0.65	0.48	0.54	0.53
Watching Television	0.61	0.49	0.49	0.46
Sitting in Public Place	0.60	0.57	0.58	0.58
Vehicle Passenger	0.68	0.55	0.64	0.56
Lying Down in Afternoon	0.66	0.44	0.59	0.63
Talking to Someone	0.45	0.45	0.42	0.35
After Lunch	0.60	0.58	0.58	0.57
Driving	0.44	0.34	0.43	0.40

*IQR-Interquartile range.

**Figure 1:**
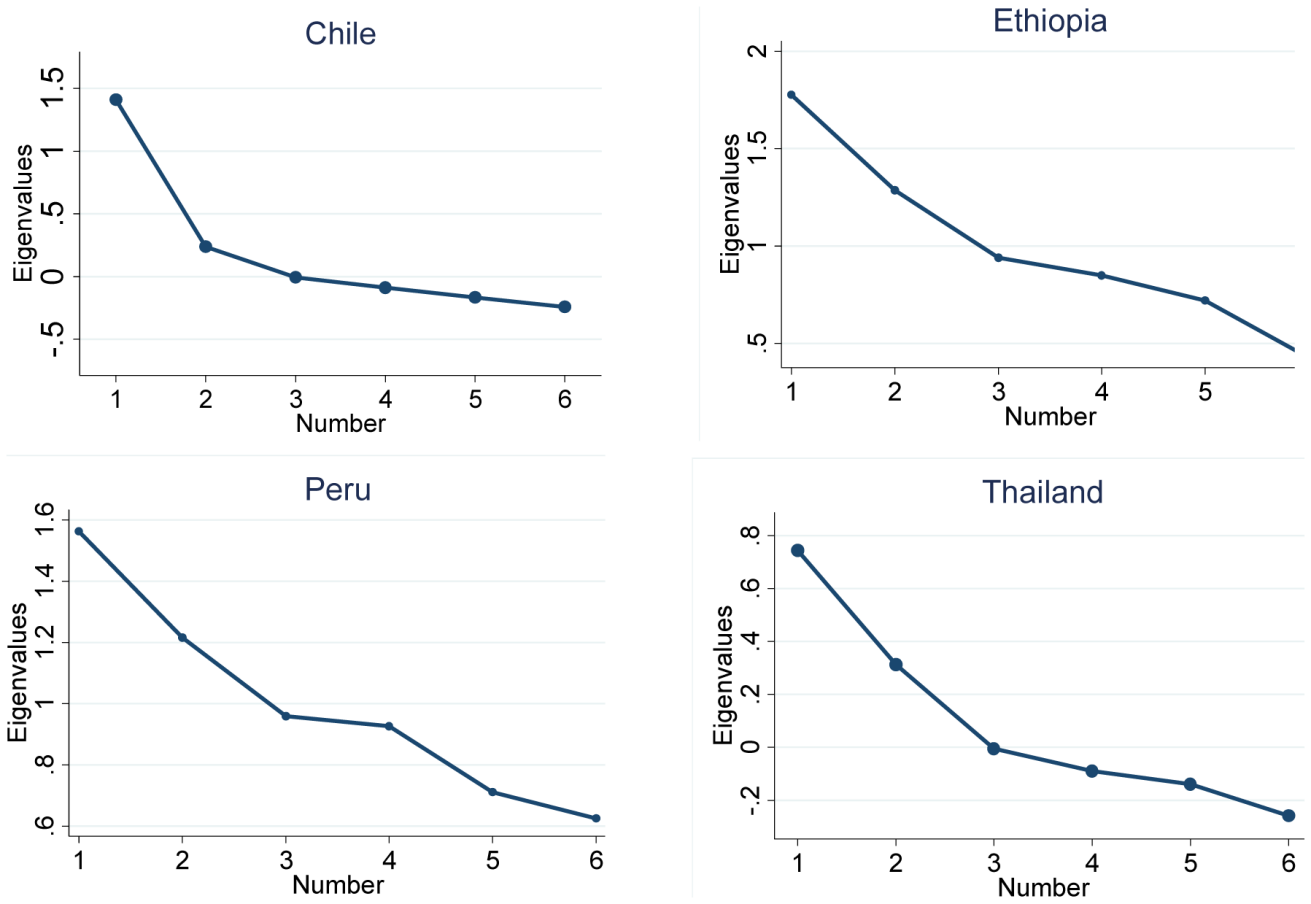
Scree plot of Pittsburgh Sleep Quality Index (PSQI) according to country.

Through examination of eigenvalues, factor loadings and the scree plot for PSQI subscales, a two-factor model was extracted for Chile, Ethiopia and Thailand (**Fig. 1**). However, a three-factor model provided a better fit for the data from Peru (Fig. 1). **Table 3** shows the principal component analysis (PCA) results for PSQI and ESS subscales according to county. Similar to the scree plots, a two-factor model of the PSQI was extracted in Chile, Ethiopia and Thailand. The first factor (quality) consisted of sleep disturbance, sleep latency, daytime dysfunction, sleep medication and sleep quality. The second factor (efficiency) consists of sleep duration and sleep efficiency. For Peru, the better fitting three-factor model was as follows: first factor (sleep quality) consists of sleep disturbance, daytime dysfunction and sleep quality; second factor (sleep efficiency) consists of sleep duration and sleep efficiency; and third factor (medication) consists of sleep latency and sleep medication. The two-factor model explained approximately 46% of the total variance in Thailand, 48% in Ethiopia, and 49% in Chile while the three factor model in Peru explained approximately 59% of the total variance.

**Table 3:** Principal component analysis for PSQI and ESS subcomponents according to country.

	Chile		Ethiopia		Peru			Thailand	
PSQI components	Factor 1	Factor 2	Factor 1	Factor 2	Factor 1	Factor 2	Factor 3	Factor 1	Factor 2
Sleep Disturbance	**0.731**	−0.064	**0.672**	0.054	**0.582**	−0.110	0.408	**0.620**	−0.090
Sleep Latency	**0.640**	0.099	**0.467**	0.169	0.308	−0.076	**0.565**	**0.673**	0.096
Daytime Dysfunction	**0.527**	0.290	**0.685**	0.006	**0.766**	0.056	−0.081	**0.547**	0.057
Sleep Quality	**0.694**	0.358	**0.694**	0.358	**0.748**	0.358	0.044	**0.701**	0.202
Sleep Medication	**0.522**	−0.010	**0.522**	−0.010	−0.073	0.104	**0.807**	**0.428**	0.061
Sleep Efficiency	0.022	**0.744**	0.056	**0.884**	−0.076	**0.796**	0.109	0.081	**0.791**
Sleep Duration	0.110	**0.825**	0.032	**0.880**	0.265	**0.770**	−0.044	0.046	**0.826**
*Variance explained (%)*	28.42	21.00	25.07	23.00	23.73	18.57	16.57	26.00	19.62
*Total variance explained (%)*	49.42		48.08		58.88		45.62		

	Chile		Ethiopia		Peru		Thailand	
ESS items	Factor 1	Factor 2	Factor 1	Factor 2	Factor 1	Factor 2	Factor 1	Factor 2
Siting/Reading	**0.650**	0.188	**0.406**	0.300	**0.635**	0.022	**0.557**	0.036
Watching Television	**0.566**	0.258	**0.490**	0.243	**0.420**	0.264	**0.326**	0.237
Public Place	0.420	**0.490**	**0.434**	0.379	0.409	**0.460**	**0.526**	**0.352**
Vehicle Passenger	**0.694**	0.136	**0.528**	0.232	**0.698**	0.099	**0.651**	−0.058
Lying Down in Afternoon	**0.746**	0.016	**0.737**	−0.275	**0.724**	−0.058	**0.726**	0.028
After Lunch	**0.566**	0.158	**0.684**	0.115	**0.506**	0.268	**0.463**	0.329
Talking to Someone	0.090	**0.787**	0.016	**0.719**	0.044	**0.774**	−0.006	**0.818**
Driving	0.080	**0.782**	−0.181	**0.705**	−0.011	**0.760**	0.081	**0.722**
*Variance explained (%)*	28.65	20.17	21.32	20.71	25.21	19.28	23.32	18.57
*Total variance explained (%)*	48.82		42.03		44.50		41.89	

The lower panel of **Table 3** shows the principal component analysis results for ESS items.

**Figure 2:**
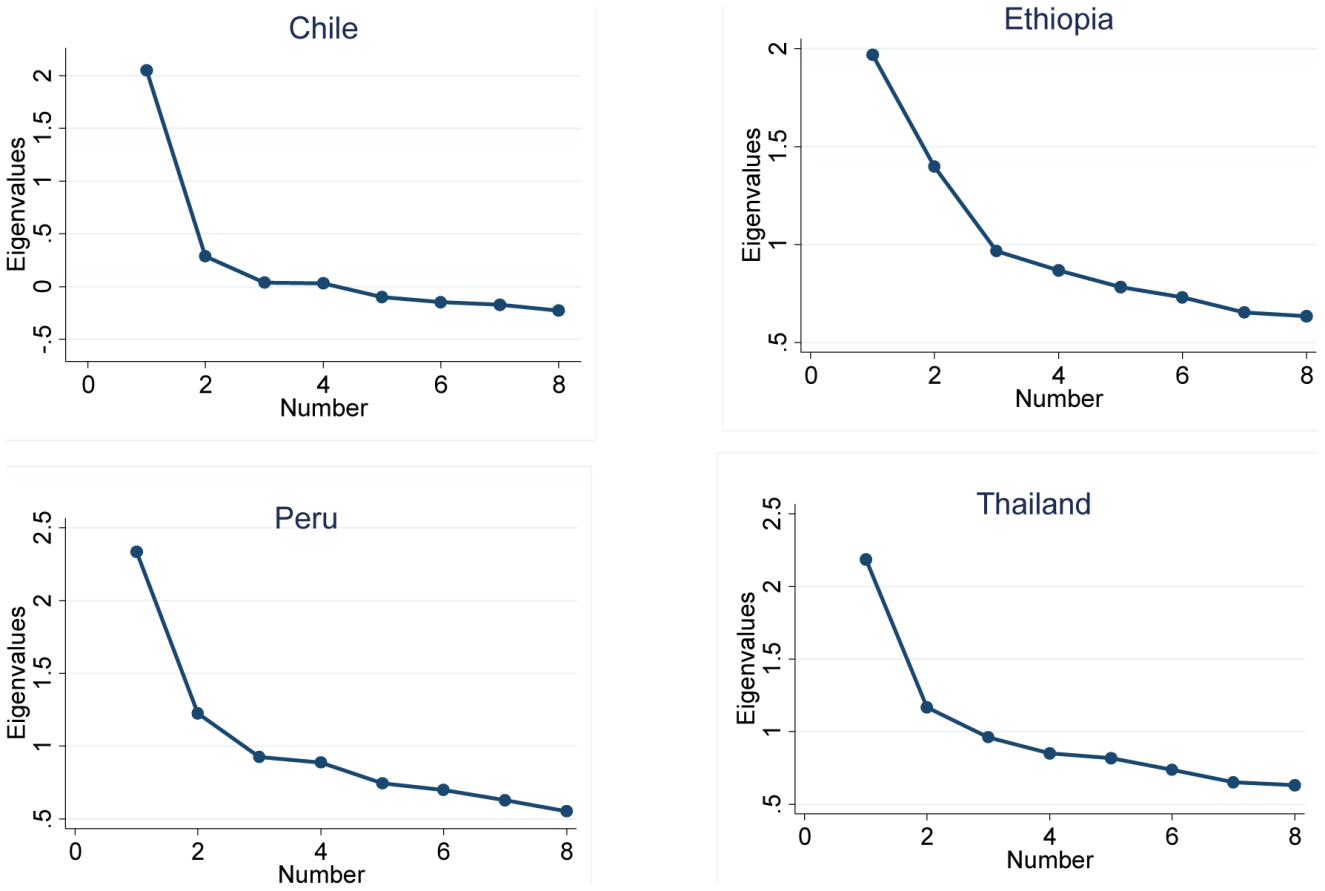
Scree plot of Epworth Sleepiness Scale (ESS) according to country.

In all four countries the ESS had two factors with eigenvalues >1.0 which explained more than 41% of the total variance (41.9% in Thailand and 48.8% in Chile). Examination of the scree plot also showed two factors (**Fig. 2**). The first factor was comprised of life situations in which dozing off is socially acceptable (sitting/reading, sitting in a public place, as a vehicle passenger, lying down in the afternoon, sitting quietly after lunch). The second factor was comprised of situations in which dozing off is socially unacceptable (talking to someone, driving).

**Figure 3:**
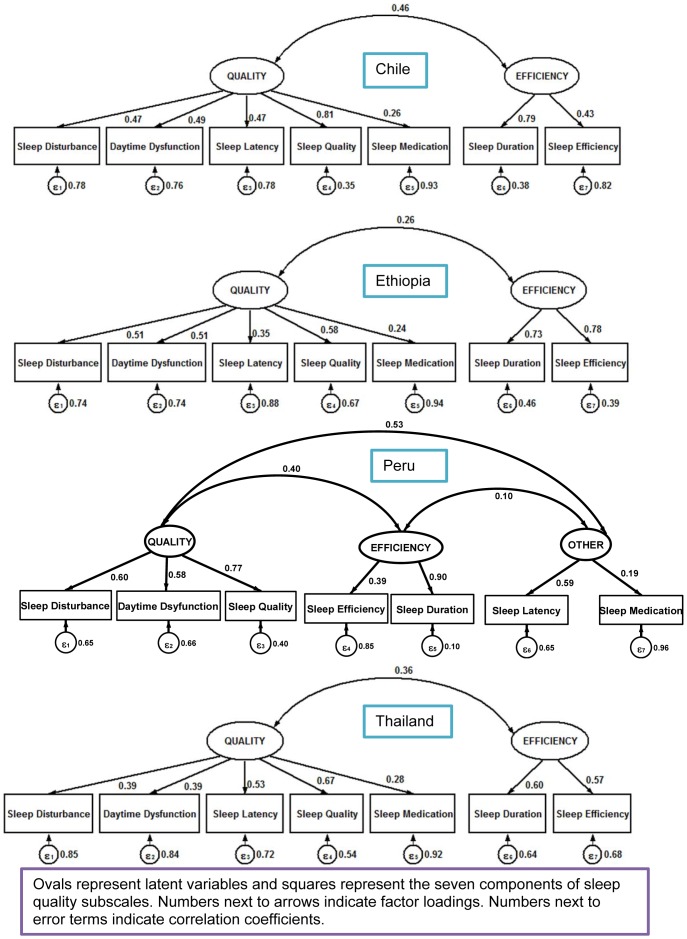
Confirmatory factor analysis of Pittsburgh Sleep Quality Index (PSQI) according to country.

**Figure 4:**
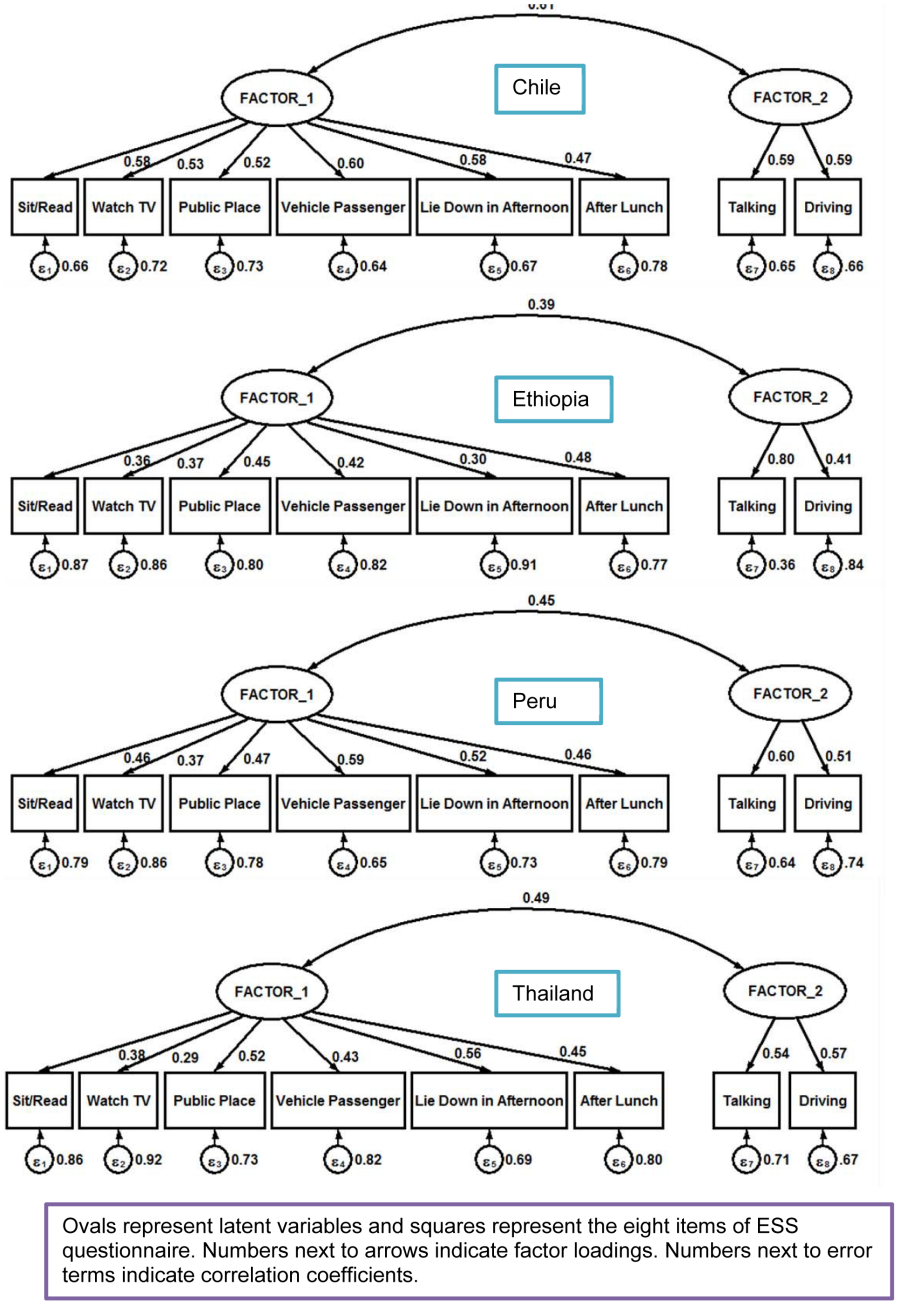
Confirmatory factor analysis of Epworth Sleepiness Scale (ESS) according to country.

In addition to our EFA, we carried out a CFA by country to examine the construct validity and to test whether the factorial structure of PSQI is comprised of two factors (**Table 4**). Adequate fit indices, indicating satisfactory model fit, were found for all the countries. In Ethiopia, Chile and Thailand the comparative fit index and Tucker-Lewis index values, indicators of goodness of fit, for PSQI were higher than 0.80. Whereas the comparative fit index and Tucker-Lewis index values for PSQI in Peru were 0.81 and 0.69 respectively. The values found suggest that a two-factor model (quality and efficiency) can be considered in the three countries while a two factor model might not be the best fit for Peru. The RMSEA values ranged from 0.042 to 0.090 indicating a good fit in Ethiopia and Thailand and a reasonably close fit in Chile and Peru. The estimates of the loadings of each observed measure on each of the factors are presented in **Fig. 3**. Similar to PSQI, as shown in lower panel of Table 4, a CFA was carried out for ESS by country (Table 4a). Overall, the results obtained from the fitted CFA model support the two-factor model for ESS. In Chile and Thailand the comparative fit index and Tucker-Lewis index values, indicators of goodness of fit, for ESS were higher than 0.86 while in Peru they were 0.85 and 0.78 respectively. Lower fit indices were found in Ethiopia (comparative fit index = 0.76 and Tucker-Lewis index = 0.64). The RMSEA values ranged from 0.056 to 0.087 indicating a good fit in Chile and Thailand and a reasonably close fit in Ethiopia and Peru. The estimates of the loadings of each observed measure on each of the factors are shown in **Fig. 4**.

**Table 4:** Confirmatory Factor Analysis for PSQI and ESS according to country.

PSQI	Chile	Ethiopia	Peru	Thailand
Items	Loadings (SE)			
***Quality***				
Sleep Disturbance	0.473 (0.035)	0.507 (0.025)	0.538 (0.023)	0.385 (0.022)
Sleep Latency	0.471 (0.034)	0.351 (0.026)	0.339 (0.025)	0.531 (0.022)
Daytime Dysfunction	0.492 (0.033)	0.512 (0.025)	0.498 (0.023)	0.394 (0.021)
Sleep Quality	0.809 (0.032)	0.578 (0.026)	0.607 (0.026)	0.675 (0.022)
Sleep Medication	0.256 (0.040)	0.249 (0.027)	0.140 (0.026)	0.276 (0.023)
***Efficiency***				
Sleep Efficiency	0.429 (0.050)	0.783 (0.052)	0.210 (0.022)	0.568 (0.041)
Sleep Duration	0.788 (0.077)	0.732 (0.049)	1.000 (0.060)	0.599 (0.042)
RMSEA	0.090	0.042	0.088	0.055
95% CI of RMSEA	(0.074, 0.107)	(0.032, 0.053)	(0.079, 0.098)	(0.046, 0.064)
CFI	0.880	0.970	0.810	0.932
TLI	0.807	0.951	0.692	0.889

ESS	Chile	Ethiopia	Peru	Thailand
Items	Loadings (SE)			
***Factor 1***				
Siting/Reading	0.584 (0.029)	0.359 (0.026)	0.461 (0.021)	0.379 (0.022)
Watching Television	0.532 (0.031)	0.368 (0.026)	0.374 (0.022)	0.290 (0.023)
Public Place	0.523 (0.031)	0.451 (0.025)	0.469 (0.022)	0.523 (0.021)
Vehicle Passenger	0.600 (0.029)	0.420 (0.025)	0.593 (0.020)	0.429 (0.021)
Lying Down in Afternoon	0.575 (0.030)	0.302 (0.028)	0.523 (0.021)	0.559 (0.021)
After Lunch	0.472 (0.033)	0.479 (0.026)	0.455 (0.022)	0.452 (0.021)
***Factor 2***				
Talking to Someone	0.594 (0.043)	0.802 (0.066)	0.596 (0.038)	0.538 (0.031)
Driving	0.585 (0.043)	0.406 (0.037)	0.507 (0.034)	0.572 (0.033)
RMSEA	0.060	0.087	0.081	0.056
95% CI of RMSEA	(0.046, 0.074)	(0.079, 0.094)	(0.073, 0.088)	(0.049, 0.063)
CFI	0.944	0.759	0.853	0.910
TLI	0.918	0.645	0.783	0.867

CFI: comparative fit index; TLI: Tucker-Lewis index; SE: Standard Error; RMSEA: Root Mean Square Error of Approximation.

## Discussion

We evaluated the construct validity and factor structures of the PSQI and the ESS in a large diverse sample of young adults. Overall our study results showed cross-cultural measurement equivalence and adequate psychometric properties regarding construct validity of PSQI and ESS in four countries. Although both the PSQI and ESS were originally developed as single-factor scales [13], [16], the results of our confirmatory and exploratory factor analyses revealed the multi- dimensionality of these questionnaires among college students.

Overall the PSQI demonstrated acceptable sub-scale-total correlations across the four countries. However poor subscale-total correlations were observed for sleep medicine use across four countries with the lowest observed in Peru (0.28). Hence, it appears that the sleep medicine subscale might be excluded when determining PSQI global scores among young adults across the four countries. Similar observation was noted by other investigators [32]. The other subscale-total correlations were 0.4 or higher across four countries, indicating that subscales measure the same underlying construct of sleep quality. The subscales with the highest subscale-total correlation were noted for sleep quality (r = 0.71) in Chile, sleep efficiency (r = 0.65) in Ethiopia, sleep duration (r = 0.64) in Peru and sleep quality (r = 0.63) in Thailand. In the present study the PSQI was noted to have two factor-structures in Chile, Ethiopia, and Thailand while a three-factor model was extracted in Peru. Although the developers of the PSQI have proposed a unidimensional structure and a single global scoring protocol for the information collected using the questionnaire, in concurrence to our study findings, several investigators have reported that in different sample populations the questionnaire might be better represented by a two [20], [34], or three [18], [19] factor structures rather than a one- factor structure. Therefore, a single summed global score resulting from the six subscales of PSQI might not best capture the multidimensional nature of poor sleep quality [35].

The ESS demonstrated good internal consistency and item-total correlations across four countries. However, items 8 and 6 (sleeping while driving when a car stopped in traffic and sleeping while talking to someone, respectively) showed the lowest item-total correlations with marked cross-country variations. The lowest item-total correlation for item 8 (in a car while stopped for a few minutes in the traffic) was the lowest in Ethiopia (0.34). This is not surprising where the vast majority of students are not drivers in Ethiopia. The lowest item-total score correlation for item 6 (sitting and talking to someone) was observed in Thailand (0.35). There is probably a cultural aspect to this item that requires further investigation. Others have reported similar observations [21]. In instances where questions may not be applicable given cultural and pragmatic reasons, some investigators have modified the specific tasks to fit the study population context. For instance, Storfer-Isser et al in their study among adolescents at Cleveland Children's Sleep and Health Study replaced the item ‘in a car while stopped for few minutes in traffic’ with ‘doing homework or taking a test’ [36]. Similar contextual modifications might be considered in future multi-national studies. The other subscale-total correlations were greater than 0.4 across four countries, indicating that subscales measure the same underlying construct of excessive daytime sleepiness.

The ESS was originally developed as single-factor model [13], [16]. However, a growing body of literature has shown that the eight items of ESS do not assess a unidimensional construct. The ESS was found to fit a two-dimensional (two factor model) across all four countries. As noted by Smith et al the eight items of the ESS do not assess a unitary ‘somnoficity’ construct in this population [21]. Hence, collectively, the results reported by Smith et al and our present results suggest that the sum of all the eight items may not likely be the best index of excessive daytime sleepiness when used in cross-cultural settings.

Using previously defined published cut points, we found high prevalence estimates of poor sleep quality and excessive daytime sleepiness across four countries (**S3 Fig.**). These results are in general agreement with the existing but limited body of literature specific to LAMICs [37], [38] with some variation across countries. The highest poor sleep quality prevalence was noted in Peru with 56%, followed by Ethiopia (53%), Chile (51.8%) and Thailand (48.1%). The highest excessive daytime sleepiness prevalence was noted in Peru with 34.6%, followed by Chile (31.3%), Thailand (27.8%) and Ethiopia (26.1%). Overall these prevalence estimates are comparable to what is reported in high-income countries [39]–[41]. These findings indicate the need for sleep promotion programs targeting young adults in LAMICs [3], [37], [38], [42], [43].

Strengths of the current study include a relatively large sample size, diverse group of participants and standard administration of study instruments. Nevertheless, this current cross-cultural study is not without limitations. Criterion validity utilizing diagnostic gold standard and test-retest reliability using repeated measures were not assessed. However, the criterion validity of PSQI and ESS has been previously shown to be appropriate when compared with more invasive measures [13], [16]. Another limitation that merits consideration is the standard cut-points used in this study to compare sleep disorders across the countries (>5 for the PSQI, >10 for the ESS). Given the multidimensionality of these scales, future studies with sleep disorder diagnoses based on clinical evaluations should evaluate the sensitivity and specificity of these cut-points for the valid assessment of clinically defined sleep disorders in younger adults. As demonstrated in this study single factor global score may not best capture the multidimensional nature of sleep disturbances. Finally, our study was restricted to young men and women enrolled in colleges or university classes. Hence, the readers should exercise caution when generalizing results from the present study to general population.

Despite variations in factor structures, data collection methods, social, geographic, racial and ethnic differences of populations studied to date, available evidence suggests that poor sleep quality and excessive daytime sleepiness are highly prevalent among college students across the globe and are an emerging important public health problems. The consequences of sleep insufficiency are far reaching. There is a need for public health professionals, educators as well as researchers to recognize the growing problem of sleep disorders in LAMICs [3], [37], [38], [42], [43]. Surveillance and monitoring of sleep insufficiency in vulnerable populations and particularly those in emerging economies globally will be important. However, in order to enhance the likelihood of collecting valid information regarding the prevalence and public health consequences of sleep insufficiency, care should be taken in assessing the utility of questionnaires such as the PSQI and ESS in cross-cultural settings.

## Supporting Information

S1 Fig **Distribution of Pittsburgh Sleep Quality Index (PSQI) total score according to country.**
(TIF)

S2 Fig **Distribution of Epworth Sleepiness Scale (ESS) total score according to country.**
(TIF)

S3 Fig **Prevalence poor sleep quality and excessive daytime sleepiness using published cut-off scores.**
(TIF)
