# Supplementary figures and images for: Construct Validity and Factor Structure of the Pittsburgh Sleep Quality Index and Epworth Sleepiness Scale in a Multi-National Study of African, South East Asian and South American College Students
Source: PLoS One. 2014 Dec 31;9(12):e116383. doi: 10.1371/journal.pone.0116383 (PMC4281247; doi:10.1371/journal.pone.0116383)

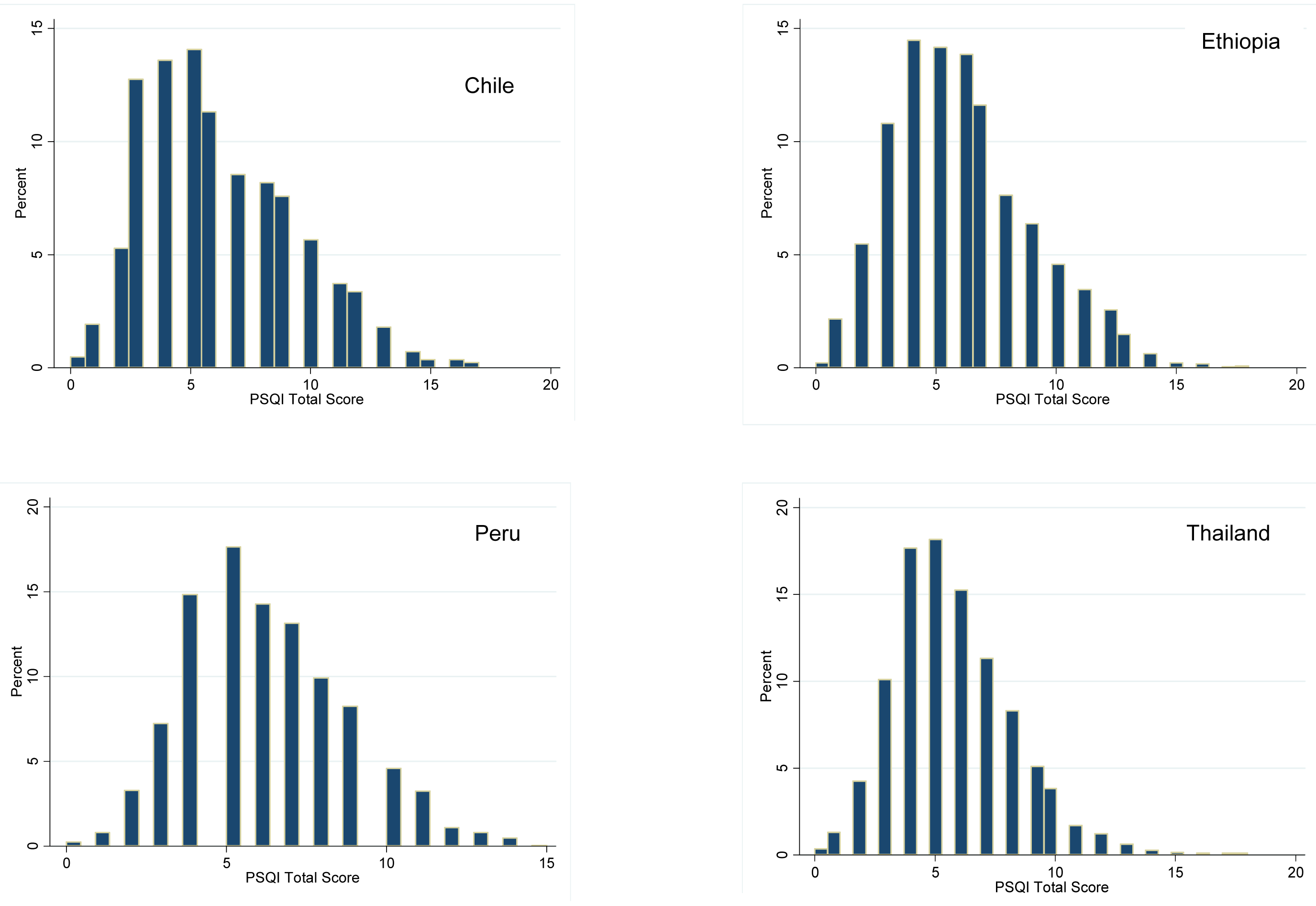

Supplement: S1 Fig — Distribution of Pittsburgh Sleep Quality Index (PSQI) total score according to country. (TIF) [file pone.0116383.s001.tif]

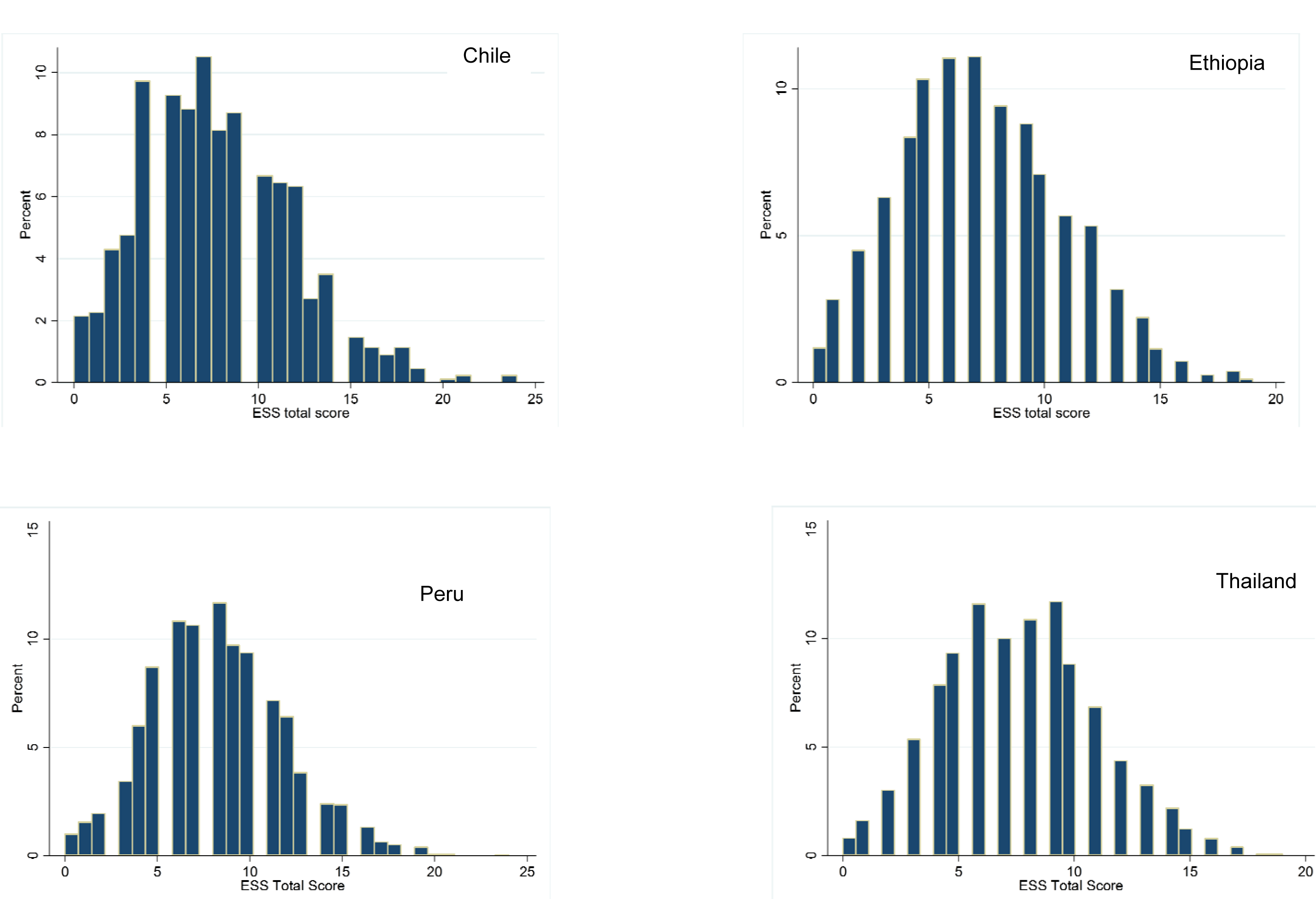

Supplement: S2 Fig — Distribution of Epworth Sleepiness Scale (ESS) total score according to country. (TIF) [file pone.0116383.s002.tif]

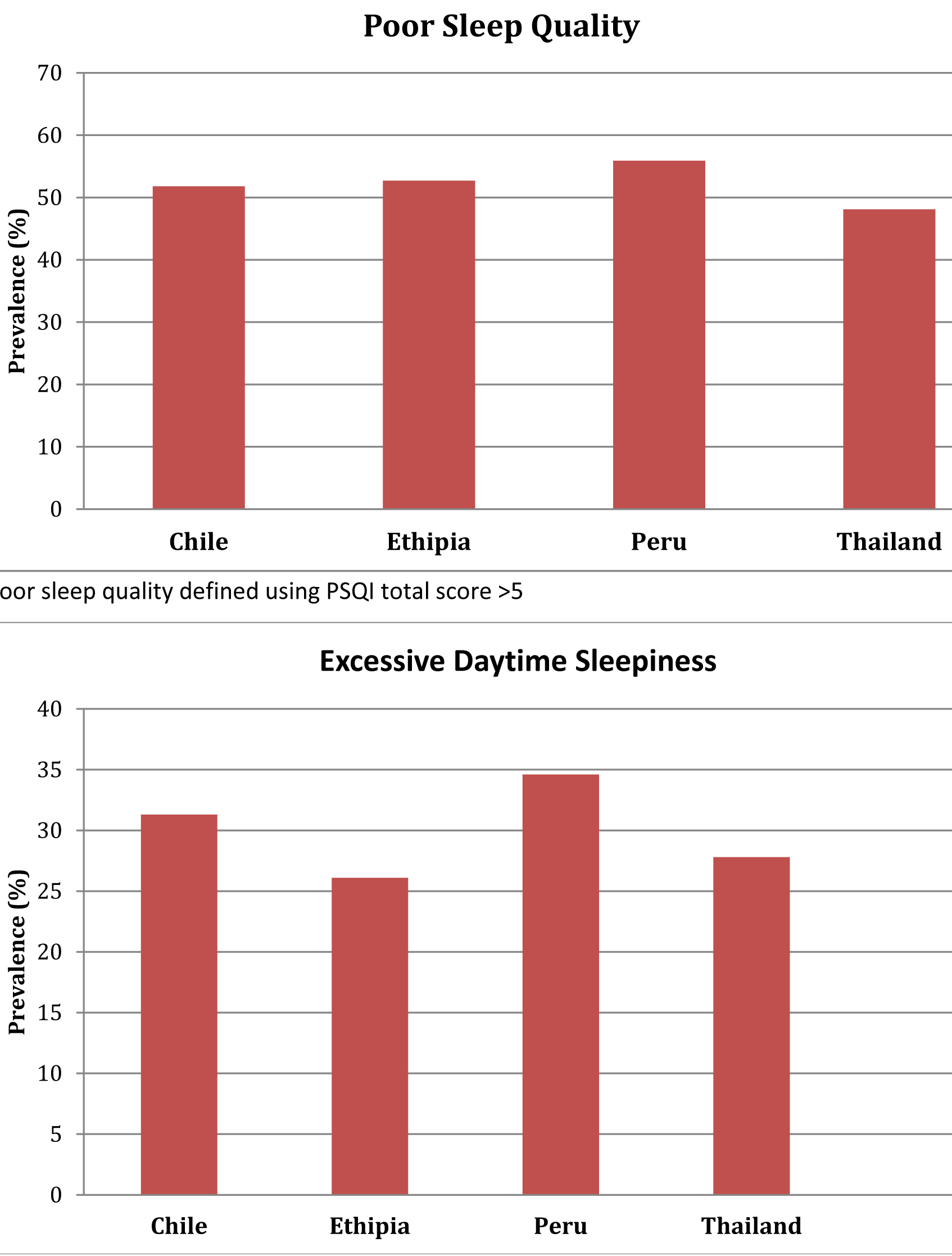

Supplement: S3 Fig — Prevalence poor sleep quality and excessive daytime sleepiness using published cut-off scores. (TIF) [file pone.0116383.s003.tif]
